# Protection against cardiac ischemia-reperfusion injury by hypothermia and by inhibition of succinate accumulation and oxidation is additive

**DOI:** 10.1007/s00395-019-0727-0

**Published:** 2019-03-15

**Authors:** M. Kohlhauer, V. R. Pell, N. Burger, A. M. Spiroski, A. Gruszczyk, J. F. Mulvey, Amin Mottahedin, A. S. H. Costa, C. Frezza, B. Ghaleh, M. P. Murphy, R. Tissier, T. Krieg

**Affiliations:** 1 U955, IMRB, Inserm, UPEC, Ecole Nationale Vétérinaire d’Alfort, Créteil, France; 2 Department of Medicine, University of Cambridge, Cambridge, CB2 0QQ UK; 3 Medical Research Council Mitochondrial Biology Unit, University of Cambridge, Cambridge, CB2 0XY UK; 4 Department of Physiology, Institute of Neuroscience and Physiology, Sahlgrenska Academy, University of Gothenburg, Gothenburg, Sweden; 5 Medical Research Council Cancer Unit, University of Cambridge, Cambridge, CB2 0XZ UK

**Keywords:** Hypothermia, Succinate, Dimethyl malonate, Myocardial infarction, Ischemia, Reperfusion

## Abstract

Hypothermia induced at the onset of ischemia is a potent experimental cardioprotective strategy for myocardial infarction. The aim of our study was to determine whether the beneficial effects of hypothermia may be due to decreasing mitochondria-mediated mechanisms of damage that contribute to the pathophysiology of ischemia/reperfusion injury. New Zealand male rabbits were submitted to 30 min of myocardial ischemia with hypothermia (32 °C) induced by total liquid ventilation (TLV). Hypothermia was applied during ischemia alone (TLV group), during ischemia and reperfusion (TLV-IR group) and normothermia (Control group). In all the cases, ischemia was performed by surgical ligation of the left anterior descending coronary artery and was followed by 3 h of reperfusion before assessment of infarct size. In a parallel study, male C57BL6/J mice underwent 30 min myocardial ischemia followed by reperfusion under either normothermia (37 °C) or conventionally induced hypothermia (32 °C). In both the models, the levels of the citric acid cycle intermediate succinate, mitochondrial complex I activity were assessed at various times. The benefit of hypothermia during ischemia on infarct size was compared to inhibition of succinate accumulation and oxidation by the complex II inhibitor malonate, applied as the pro-drug dimethyl malonate under either normothermic or hypothermic conditions. Hypothermia during ischemia was cardioprotective, even when followed by normothermic reperfusion. Hypothermia during ischemia only, or during both, ischemia and reperfusion, significantly reduced infarct size (2.8 ± 0.6%, 24.2 ± 3.0% and 49.6 ± 2.6% of the area at risk, for TLV-IR, TLV and Control groups, respectively). The significant reduction of infarct size by hypothermia was neither associated with a decrease in ischemic myocardial succinate accumulation, nor with a change in its rate of oxidation at reperfusion. Similarly, dimethyl malonate infusion and hypothermia during ischemia additively reduced infarct size (4.8 ± 2.2% of risk zone) as compared to either strategy alone. Hypothermic cardioprotection is neither dependent on the inhibition of succinate accumulation during ischemia, nor of its rapid oxidation at reperfusion. The additive effect of hypothermia and dimethyl malonate on infarct size shows that they are protective by distinct mechanisms and also suggests that combining these different therapeutic approaches could further protect against ischemia/reperfusion injury during acute myocardial infarction.

## Introduction

Acute myocardial infarction (AMI) is a leading cause of cardiovascular death, and the primary cause of chronic heart failure worldwide [4]. Percutaneous coronary intervention (PCI) reduces infarct size with early intervention and markedly improves long-term clinical outcomes. However, PCI-mediated tissue reperfusion of ischemic tissue in itself causes irreversible myocardial damage due to reperfusion injury [12]. Therefore, reduction of both ischemia- and reperfusion-mediated tissue injuries represents key opportunities for new therapies [13].

The induction of mild hypothermia of 32–34 °C at the onset of ischemia that is then maintained throughout reperfusion provides effective cardioprotection in experimental models of AMI [5, 10, 28]. Yet, induction of cooling at reperfusion alone does not reduce infarct size, both in experimental [11, 21, 28] and clinical settings [9]. This suggests that hypothermic cardioprotection is related to pathways specifically associated with ischemia, rather than during reperfusion. Recently the accumulation of myocardial succinate has been described as a specific and universal signature of ischemia in animals and human [3, 19, 29] that then drives reperfusion injury upon its oxidation during reperfusion. Thus, an appealing potential cardioprotective mechanism of hypothermia during ischemia is to decrease the extent of succinate accumulation during ischemia and thereby lessen tissue injury upon reperfusion.

The aim of our study was to investigate the consequences of hypothermia on the ischemic accumulation of succinate and to see if any such effects could contribute to the protection against ischemia/reperfusion injury by cooling. To isolate the effects of hypothermia on ischemia alone, we compared rabbits cooled throughout ischemia-reperfusion to those cooled during ischemia only and then very rapidly warmed to normothermia upon the onset of reperfusion. To do this, we used total liquid ventilation (TLV) of the lungs with perfluorocarbon (PFC), which enables us to effectively instantly adjust body temperature [15, 20, 28]. We also used a mouse model of myocardial ischemia with hypothermia during both ischemia and reperfusion. In all the cases, we investigated the role of hypothermia on succinate accumulation and oxidation.

We found that hypothermia during ischemia alone led to a potent reduction of cardiac infarct size in rabbits, while cooling during ischemia and reperfusion, which was protective in mice. This hypothermic cardioprotection was not due to any change in succinate accumulation during ischemia. Furthermore, the cardioprotection afforded by cooling was additive with that brought about by the pharmacological inhibition of succinate accumulation and oxidation.

## Methods

### Acute myocardial infarction in the rabbit

**Fig. 1:**
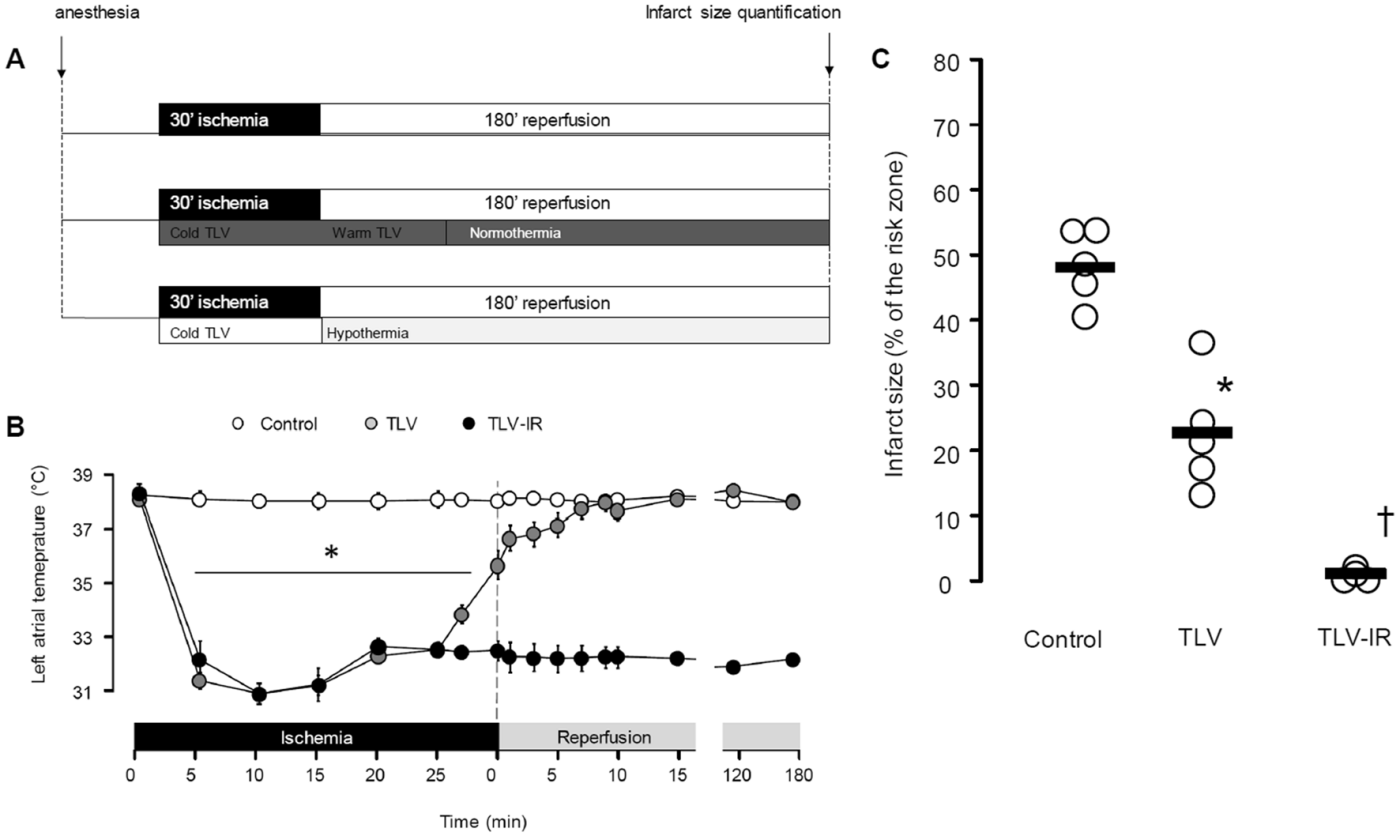
Experimental protocol in rabbits (**a**), real-time temperature regulation (**b**) and infarct size after discrete or continuous hypothermia (**c**). TLV, total liquid ventilation. *p < 0.05 vs Control; †*p* < 0.05 vs TLV and Control

All rabbit experiments were approved by the institutional review board for animal research (ComEth AnSES/ENVA/UPEC n°16), in accordance with French regulations for animal experimentation. Male New Zealand white rabbits (2.5–3.5 kg) were randomly allocated to one of six experimental groups (Figs. 1a and 3c): two normothermic groups (Control), hypothermic ischemia with normothermic reperfusion (TLV), hypothermic ischemia and hypothermic reperfusion (TLV-IR), intravenous dimethyl malonate (Malonate), and hypothermia during ischemia only with dimethyl malonate (TLV + Malonate). Five animals were included in each control group, seven in TLV, five in Malonate, four animals in TLV + Malonate and four animals in the TLV-IR group. Rabbits were anesthetized with intravenous zolazepam, tiletamine and sodium pentobarbital (all 20–30 mg/kg). Following intubation and mechanical ventilation, a surgical plane of anesthesia was maintained with sodium pentobarbital (5–10 mg/kg/h). The electrocardiogram was continuously recorded (HEM 3.5, Notocord, Croissy-sur-Seine, France), and temperature was monitored via a probe inserted in the left atrium. Following a left thoracotomy, a ligature was passed around a prominent branch of the left coronary artery, as previously described [28]. Malonate and TLV + Malonate groups received an intravenous infusion of dimethyl malonate in saline (10 mg/kg/min; 20 mg/ml), 10 min before coronary artery occlusion which continued throughout ischemia. Other groups received a comparable volume of saline. All animals underwent 30 min coronary artery occlusion followed by 180 min reperfusion. Hearts were stained with Evan’s blue dye and 2% triphenyltetrazolium chloride, as previously described [28]. This method represents the gold standard of infarct size evaluation with high short-term reliability as compared to classical histology. In additional rabbits, ischemic left ventricular tissue was collected under control and hypothermic conditions and freeze-clamped in liquid nitrogen at the end of ischemia, and following 5 min reperfusion for succinate and adenosine nucleotide quantification.

### Total liquid ventilation

Hypothermia in rabbits was induced with a pediatric preclinical liquid ventilator (Inolivent) [22]. Tidal volume was maintained at 8 mL/kg, respiratory frequency at eight breaths per minute, and positive end expiratory pressure at 5 mmHg, as previously described [20]. Perfluorocarbon (PFC) TLV was induced at the onset of ischemia, and for 30 min during reperfusion. PFC temperature was initially set to 21 °C for induction of cooling, and progressively adjusted to 32 °C for temperature stabilization. Five minutes before reperfusion, PFCs were rewarmed to 40 °C to induce ultra-fast (< 120 s) rewarming at the onset of reperfusion. Liquid ventilation was then continued for an additional 30 min to stabilize temperature prior to resumption of conventional gaseous ventilation (Fig. 1a).

### Acute myocardial infarction in the mouse

All mouse experiments were carried out in accordance with the UK Animals (Scientific Procedures) Act 1986 and the University of Cambridge Animal Welfare policy (Project license 70/8238).

**Fig. 2:**
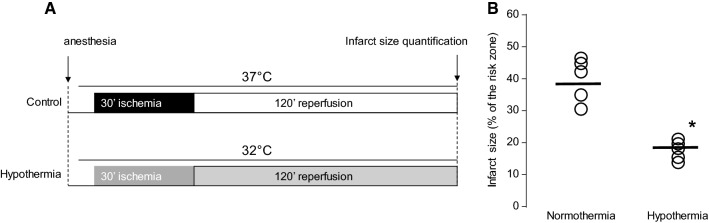
Experimental protocol (**a**) and infarct size (**b**) measurements in mice. *p <* 0.05* vs Control

Male mice (8–10 weeks; C57BL/6J; Charles River Laboratories) were randomly allocated to either normothermic (37 °C) or hypothermic (32 °C) groups with five mice in each group. In the hypothermic group, mice were cooled to 32 °C throughout the entire experiment, using cutaneous application of ice packs (Fig. 2). Mice were anesthetized with 70 mg/kg intraperitoneal sodium pentobarbital, intubated endotracheally, and ventilated at 110 breaths per minute (tidal volume 125–150 µL, dependent on weight). Core temperature was monitored continuously via a rectal probe. A sternal thoracotomy was performed, and the major branch of the left anterior descending coronary artery was occluded for 30 min as previously described [3]. After ischemia, myocardial reperfusion was induced by releasing the coronary suture for 120 min. Hearts were stained with Evan’s blue dye and 2% triphenyltetrazolium chloride. The infarct area was analyzed as a percentage of the risk zone, as previously described [3].

In additional mice, ischemic and non-ischemic left ventricular tissue was collected at the end of 30 min ischemia or following 1, 2 and 5 min of reperfusion. Tissues were snap-frozen in liquid nitrogen or frozen with a clamp cooled in liquid nitrogen for metabolite quantification and stored at — 80 °C.

### Reverse electron transfer

Hearts were retrieved from 10 to 12 weeks Wistar rats, heart tissue was homogenized in STEB (250 mM sucrose, 5 mM Tris–HCl, 1 mM EGTA, 0.1% fatty acid-free BSA, pH 7.4, 4 °C) and centrifuged for the isolation mitochondria by differential centrifugation at 4 °C (2 × 700 *g* for 5 min, 2 × 10.000 *g* for 10 min). The protein content of isolated mitochondria was measured by the bicinchonic acid assay following the standard protocol. Superoxide production by reverse electron transfer (RET) was assessed at either 32 °C or 37 °C by measuring the H_2_O_2_ efflux in KCl buffer (120 mM KCl, 10 mM HEPES, 1 mM EGTA, pH 7.2) containing 5 mmol/L succinate and fatty acid-free BSA (0.2 mg/ml). The H_2_O_2_ concentration was measured by the oxidation of Amplex Red (12.5 µM/L, Invitrogen) to resorufin in the presence of superoxide dismutase (100 units/ml) and horseradish peroxidase (4 units/ml). Resorufin was detected using *λ*_ex_ = 560 nm and *λ*_em_ = 590 nm with a CLARIOstar microplate reader (BMG Labtech).

The efflux was calibrated using freshly prepared H_2_O_2_ linear standard curves (*ε*_240 nm_ = 43.5 M^−1^cm^−1^). The standard curves were measured with each assay in the presence of all buffer components and mitochondria, lacking only succinate.

### Metabolite quantification

Succinate, hypoxanthine and xanthine levels were quantified in mouse myocardium and succinate in rabbit myocardium by liquid chromatography-mass spectrometry (LC–MS). Briefly, 10 mg tissue was lysed in 250 μL extraction solution (30% acetonitrile, 50% methanol, and 20% water). The suspension was immediately centrifuged (16,000 *g*, 15 min at 0 °C), and the supernatant was used for LC–MS analysis. After liquid chromatography, the mass spectrometer (Thermo QExactive Orbitrap) was operated in full MS and polarity switching mode. Samples were randomized to avoid bias due to machine drift. Absolute quantification of metabolites was performed by interpolation of the corresponding standard curve obtained from serial dilutions of a reference standard (Sigma Aldrich) run concurrent with the samples. Xanthine and hypoxanthine concentrations were expressed as x-fold relative to non-ischemic myocardium.

ATP and ADP concentrations were measured using a luciferase-based assay (Strehler, 1974). Briefly, frozen tissue samples were homogenized in ice-cold perchloric acid extractant (3% v/v HClO_4_, 2 mM Na_2_EDTA, 0.5% Triton X-100). The supernatant was diluted to a concentration of 1 mg frozen tissue/ml. Samples, ATP and ADP standards (400 µl) were pH neutralized using a potassium hydroxide solution (2 M KOH, 2 mM Na_2_EDTA, 50 mM MOPS). For ADP measurements, 250 µl neutralized sample supernatant was mixed with 250 µl ATP sulfurylase assay buffer [20 mM Na_2_MoO_4_, 5 mM GMP, 100 mM Tris–HCl, 10 mM MgCl_2_, 0.2 U ATP sulfurylase (New England Biolabs)], incubated for 30 min at 30 °C, heated to 100 °C for 5 min and then cooled on ice. Standards (100 µl), samples for ATP measurement (100 µl) or samples for ADP measurement (200 µl) were added to 400 µl Tris–acetate (TA) buffer (100 mM Tris, 2 mM Na2EDTA, 50 mM MgCl_2_) in luminometer tubes. 10 µl pyruvate kinase solution [100 mM PEP, 6 U pyruvate kinase suspension (SIGMA)] was added to samples for ADP measurement and incubated for 30 min at 25 °C in the dark to convert ADP to ATP. The samples were then assayed for ATP content in a Berthold AutoLumat Plus luminometer by addition of 100 µl luciferase/luciferin Solution [7.5 mM DTT, 0.4 mg/ml BSA, 1.92 µg luciferase/ml (SIGMA), 120 µM d-luciferin (SIGMA), made in TA buffer (25% v/v glycerol)]. Bioluminescence of the ATP-dependent luciferase activity was measured post injection and the data quantified against standard curves.

### Statistical analysis

Statistical analyses were conducted with Systat^®^ 13.1 (Systat Software Inc.). Comparisons were made using one-way analysis of variance (ANOVA) with Bonferroni correction. Data in Fig. 1b were compared using two-way ANOVA followed by Bonferroni correction accordingly. Values are expressed as means ± SEM, and significance set at *p *< 0.05.

## Results

### Hypothermia during ischemia alone reduces infarct size

To assess the effect of hypothermia during ischemia only, followed by normothermia upon reperfusion, we used TLV to cool rabbits to 32 °C during ischemia and also to warm them rapidly to 37 °C at the onset of reperfusion (Fig. 1b). This led to a significant decrease of infarct size compared to control. Additionally, rabbits cooled by the same method and maintained cool during reperfusion showed a significant decrease of infarct size compared to discrete hypothermia only during ischemia (2.8 ± 0.6%, 24.9 ± 3.0% and 49.6 ± 2.6% of the area at risk, in TLV-IR, TLV and Control groups, respectively; Fig. 1c). This was consistent with the effect of cooling on mice maintained at 32 °C during ischemia and reperfusion, which showed a significant decrease of infarct size in hypothermic mice as compared to normothermic controls (16.0 ± 1.9% vs. 39.8 ± 3.7%, respectively; Fig. 2b). For technical reasons it was not possible to cool mice during ischemia and then rapidly warm them at the onset of reperfusion. These findings suggested that cooling during ischemia is sufficient to decrease IR injury, and that cooling during both ischemia and reperfusion gives greater protection than cooling during ischemia only.

Hypothermia does not prevent succinate accumulation during ischemia or its rapid oxidation at reperfusion

**Fig. 3:**
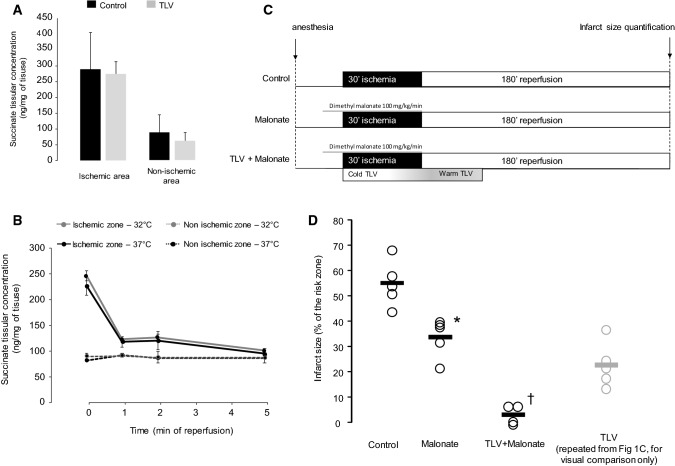
Tissue concentrations of succinate at the end of ischemia in non-ischemic and ischemic area in rabbit (**a**), myocardial concentration of succinate during ischemia and reperfusion in mice, in ischemic and non-ischemic areas (**b**), experimental protocol for evaluation of succinate inhibition by dimethyl malonate in rabbits with or without discrete hypothermia (**c**) and subsequent infarct size (**d**). TLV, total liquid ventilation. *p < 0.05 vs Control; †*p* < 0.05 vs Malonate group

As succinate accumulation during ischemia drives ROS production upon reperfusion, one possibility is that hypothermia is protective by reducing succinate accumulation during ischemia and/or slowing its oxidation during reperfusion. To assess this possibility, we first measured the impact of discrete hypothermia on succinate accumulation during ischemia in rabbits. We found no changes in succinate levels after 30 min of hypothermic ischemia (287 ± 127 ng/mg and 267 ± 62 ng/mg of tissue in TLV and Control respectively, Fig. 3a). This was consistent with mouse experiments that showed similar succinate accumulation during hypothermic and normothermic ischemia (Fig. 3b). Furthermore, the mouse model enabled us to assess whether cooling affected the oxidation of myocardial succinate during the first minutes of reperfusion. This analysis showed that after 5 min of reperfusion, succinate levels were close to non-ischemic values for both normothermic and hypothermic mice (1.23 ± 0.07 and 1.18 ± 0.02 fold, respectively, relative to non-ischemic tissue) (Fig. 3b) suggesting that the rate of oxidation during this short timeframe upon reperfusion is not affected by cooling.

### Inhibition of succinate accumulation during hypothermic ischemia additively reduces infarct size

To see if succinate contributed to IR injury in the rabbit model, we next assessed the effect of dimethyl malonate infusion and found that it significantly decreased infarct size compared with control (Fig. 3d), suggesting that succinate accumulation during ischemia and oxidation upon reperfusion does contribute significantly to IR injury in rabbit. Interestingly, combination of both cardioprotective strategies (discrete hypothermia during ischemia only and dimethyl malonate) was significantly more effective at reducing infarct size (4.8 ± 2.2%) than each intervention alone, indicating additive, independent mechanisms (Fig. 3d).

### Mechanisms of protection by hypothermia

**Fig. 4:**
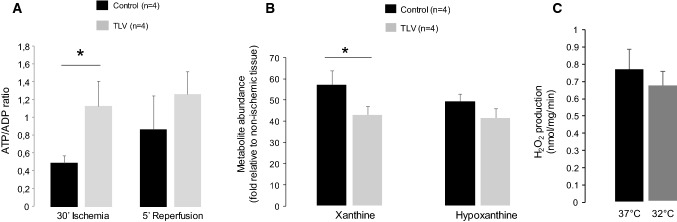
Consequence of hypothermia on ATP/ADP preservation at the end of ischemia in rabbits (**a**), on hypoxanthine and xanthine levels at the end of ischemia in mice (**b**) and on H_2_O_2_ production by reverse electron transport at 37 °C or 32 °C (**c**). *p <0.05 between groups

We next assessed the effect of hypothermia on the ATP/ADP ratio in rabbits and found a preservation of this ratio in TLV-induced hypothermia as compared to control at the end of ischemia (0.49 ± 0.08 vs 1.14 ± 0.28, respectively). There were no differences in ATP/ADP ratio after 5 min of reperfusion between the different groups (*p* = 0.45) (Fig. 4a). Mouse experiments also showed that there was less xanthine accumulation during ischemia at 32 °C as compared to normothermia (Fig. 4b). This is consistent with less adenine nucleotide breakdown during hypothermia.

As illustrated in Fig. 4d, no significant differences were observed in H_2_O_2_ production by reverse electronic transport (RET) assessed in isolated rat heart mitochondria at 32 °C versus 37 °C, suggesting that the change in temperature was not protective through decreasing the generation of ROS at complex I by RET.

## Discussion

Mild therapeutic hypothermia during ischemia is cardioprotective, even when followed by normothermic reperfusion. All previous studies investigating the role of hypothermic cardioprotection have demonstrated that hypothermia did not reduce infarct size when induced at the onset of reperfusion [11, 21, 28]. This suggests that hypothermia should be instituted during ischemia. To our knowledge, our study is the first to investigate the role of discrete hypothermia during ischemia only, followed by normothermic reperfusion. Indeed, all previous studies demonstrating hypothermic cardioprotection have investigated hypothermia induced during both ischemia and reperfusion. Our ability to address this technical limitation was only possible due to an original method of thermal body control by TLV. This method allowed us to separate the effects of cooling during ischemia from those during reperfusion. Importantly, the benefit described here is probably not related to a non-specific effect of the TLV procedure itself, but rather to hypothermia, as previous reports showed that normothermic TLV does not reduce infarct size [28].

Hypothermia during ischemia was sufficient to produce a cardioprotective effect, presumably through inhibition of alterations that contribute to IR injury that take place during ischemia. The level of cardioprotection afforded by hypothermia during ischemia only was less than that for cooling during both ischemia and reperfusion. This is consistent with previous experimental reports with conventional hypothermia [2, 21] or with TLV-mediated hypothermia [26, 28] showing almost maximal cardioprotection by cooling during both ischemia and reperfusion. The lesser benefit observed in the hypothermic group could in principle be a consequence of the slight increase in myocardial temperature just before reperfusion. However, we fell that this explanation is unlikely, due to the very short duration of this episode. Thus, hypothermia during reperfusion is protective against infarct size, but only after hypothermic ischemia. This may partly explain the failure of a recent clinical trial on hypothermic cardioprotection in ST-elevation myocardial infarction. In the clinical setting, the induction of therapeutic hypothermia only occurs close to the end of ischemia and the target temperature is rarely reached before reperfusion. For example, aggressive cooling as performed in the CHILL-MI trial, only achieved a core body temperature of 35 °C in 76% of the patients at the time of PCI [9]. Accordingly, the lack of a difference in infarct size between normothermic and hypothermic patients is likely to be because of the short duration of hypothermic ischemia. Even so, this trial demonstrated that hypothermia decreased the occurrence of heart failure, in addition to its direct effect on infarct size reduction [9]. The mechanism of this benefit is unknown, but experimental evidence shows that hypothermia induced after the onset of reperfusion can still provide positive effects independent of infarct size reduction [6]. The mechanism is likely to be a consequence of a reduction of the chronic inflammatory response [25], the no-reflow phenomenon [6, 10, 11], or of left ventricular remodeling [7], all of which are known to further worsen the prognosis following myocardial infarction [14]. These findings suggest two distinct mechanisms of hypothermic protection: direct cardioprotection during ischemia, followed by inhibition of the damaging indirect consequences of myocardial ischemia during reperfusion, i.e., no-reflow and/or occurrence of heart failure. Yet, hypothermia during reperfusion seems to exert a synergistic benefit with ischemic hypothermia on infarct size, despite being inefficient when induced alone. While there is as yet no mechanistic hypothesis to explain this paradoxical finding, it is likely that the effect of cooling on no-reflow, hemorrhage or local inflammation may contribute to further decrease in the infarct size.

Succinate accumulates during ischemia and its oxidation upon reperfusion is able to induce cell death. We have previously shown in a mouse model of myocardial ischemia that the succinate accumulated during ischemia is rapidly oxidized by succinate dehydrogenase upon reperfusion [3]. This results in reverse electron transport and in the extensive production of superoxide by complex I. Succinate accumulation is thus a key mediator of ischemia-reperfusion injury and its inhibition of its accumulation during ischemia is cardioprotective [3]. Here, we found that hypothermia does not slow succinate accumulation during ischemia or its rapid oxidation during the first minutes of reperfusion. Additionally, we compared the benefit afforded by simultaneous hypothermia and malonate infusion. Malonate has previously been shown to be protective in mice when administered as a dimethyl ester before ischemia [3]. Furthermore, it has been shown that disodium malonate is protective when administered at reperfusion [29]. When infused prior to ischemia, dimethyl malonate reduced succinate accumulation to levels comparable with non-ischemic animals [1, 3]. Thus, under these circumstances the cardioprotective benefit of malonate is close to the maximal possible benefit of succinate pathway inhibition. The fact that the protection afforded by malonate and hypothermia are additive then indicates that they act on two distinct pathways. These results seem to be in line with previous findings from our group, investigating the role of succinate accumulation during ischemic preconditioning [23]. Similarly, the cardioprotective effect of ischemic preconditioning was not explained by succinate pathway inhibition.

Hypothermia has previously been shown to be able to reduce the production of reactive oxygen species in isolated cardiomyocytes and oxidative stress measured in vivo after ischemia and reperfusion [26]. One explanation could be that hypothermia is able to mitigate the superoxide production by complex I at reperfusion. However, the extent of hypothermia used here did not affect the extent of ROS production by reverse electronic transport in heart mitochondria (Fig. 4c). The effects of hypothermia on forward electron transport at complex I have not been tested and warrant further investigation. This suggests that the effect of hypothermia on ROS production by complex I does not contribute to cardioprotection by hypothermia. Another possibility is that hypothermia preserves energy stores during ischemia by reducing energy consumption by the cells. Consistent with this, we showed that hypothermia did preserve the ATP/ADP ratio toward the end of ischemia in rabbits; however, this putative benefit may not be fully explained by hypothermia alone [27]. For example, although cold cardioplegia at 4 °C has been shown to preserve energy, mild hypothermia to 32 °C has been shown to be insufficient to promote energy preservation as compared to the extent of the cardioprotective benefit [17]. Even if a clear linear relationship has been demonstrated between myocardial temperature and infarct size in experimental models, the metabolic reduction does not seem to correlate linearly with temperature [18]. Metabolic inhibition by mild hypothermia is poor even if cardioprotection seem to be maximal at 32 °C, suggesting that metabolic reduction only partially explains the benefit of mild hypothermia. Previous studies showed that the beneficial effect on infarct size by hypothermia at 35 °C was partly mediated by activation of extracellular signal-regulated kinase (ERK) [30]. Yet, in that study, cardioprotection was evident after 20 min of hypothermic reperfusion and the specific effect of hypothermia on ERK activity during ischemia was not investigated. Another study performed on isolated cells with hypothermia induced during the reperfusion phase also demonstrated that hypothermia was associated with an increase in phosphorylated Akt and enhanced phosphorylation of Heat Shock protein 27 (HSP27) [24]. Finally, hypothermia could be protective via a direct inhibitory effect on the activity of cyclophilin D in inducing the mitochondrial transition permeability pore [16], and thereby protect against cell death upon reperfusion.

We have demonstrated that hypothermia is cardioprotective during ischemia independently of any direct effects of cooling itself on pathological pathways associated with reperfusion. This hypothermic cardioprotection during ischemia was independent of inhibiting succinate accumulation during ischemia, or its rapid consumption upon reperfusion. Although the mechanistic pathways contributing to hypothermic cardioprotection are not fully elucidated, this study presents a new approach in combining distinct pharmacological and mechanical therapeutic strategies to improve additively the clinical outcome. Such combinations of therapeutic solutions could represent the future of therapeutic approaches in myocardial infarction [8]. During acute myocardial ischemia, pharmacological compounds are unable to reach the compromised tissues due to limited blood flow; however, combination therapy with a physical intervention such as therapeutic hypothermia could provide a new clinical avenue for early intervention. Thus, induction of hypothermia prior to percutaneous coronary intervention, in combination with a complementary pharmacologic therapy upon reperfusion, could provide the best opportunity to improve outcome for patients suffering an acute myocardial infarction.
