# Correction to: Protection against cardiac ischemia-reperfusion injury by hypothermia and by inhibition of succinate accumulation and oxidation is additive

**DOI:** 10.1007/s00395-019-0731-4

**Published:** 2019-04-09

**Authors:** M. Kohlhauer, V. R. Pell, N. Burger, A. M. Spiroski, A. Gruszczyk, J. F. Mulvey, Amin Mottahedin, A. S. H. Costa, C. Frezza, B. Ghaleh, M. P. Murphy, R. Tissier, T. Krieg

**Affiliations:** 1 U955, IMRB, Inserm, UPEC, Ecole Nationale Vétérinaire d’Alfort, Créteil, France; 2 Department of Medicine, University of Cambridge, Cambridge, CB2 0QQ UK; 3 Medical Research Council Mitochondrial Biology Unit, University of Cambridge, Cambridge, CB2 0XY UK; 4 Department of Physiology, Institute of Neuroscience and Physiology, Sahlgrenska Academy, University of Gothenburg, Gothenburg, Sweden; 5 Medical Research Council Cancer Unit, University of Cambridge, Cambridge, CB2 0XZ UK

## Correction to: Basic Research in Cardiology (2019) 114:18 10.1007/s00395-019-0727-0

The original version of this article unfortunately contained a mistake.

Owing to software incompatibility in the production process, Fig. 1b was rendered incorrectly in the original publication. The corrected Fig. 1 is given below.

**Fig. 1:**
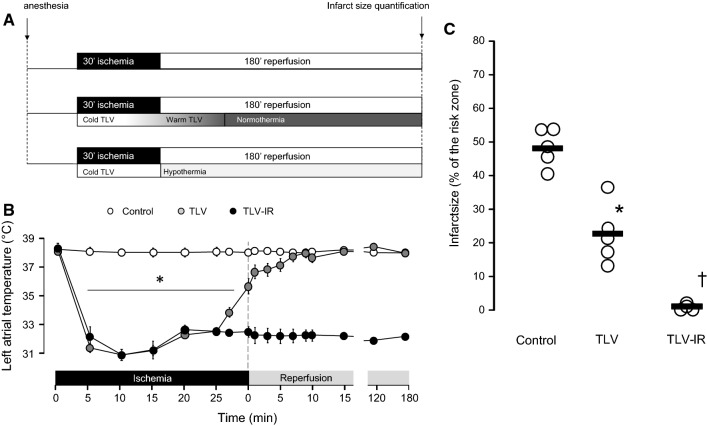
Experimental protocol in rabbits (**a**), real-time temperature regulation (**b**) and infarct size after discrete or continuous hypothermia (**c**). *TLV* total liquid ventilation. *p < 0.05 vs Control; ^†^*p *< 0.05 vs TLV and Control

The original article has been corrected.

